# Patient Rationales Against the Use of Patient-Accessible Electronic Health Records: Qualitative Study

**DOI:** 10.2196/24090

**Published:** 2021-05-28

**Authors:** Hanne Støre Valeur, Anne Kveim Lie, Kåre Moen

**Affiliations:** 1 Institute of Health and Society University of Oslo Oslo Norway

**Keywords:** patient-accessible electronic health records, open notes, active patients, patients’ perspective, patient portal, electronic health records, participation

## Abstract

**Background:**

Patient-accessible electronic health records (PAEHRs) enable patients to access their health records through a secure connection over the internet. Although previous studies of patient experiences with this kind of service have shown that a majority of users are positive toward PAEHRs, little is known about why some patients occasionally or regularly choose not to use them. A better understanding of why patients may choose not to make use of digital health services such as PAEHRs is important for further development and implementation of services aimed at having patients participate in digital health services.

**Objective:**

The objective of the study was to explore patients’ rationales for not embracing online access to health records.

**Methods:**

Qualitative interviews were conducted with 40 patients in a department of internal medicine in a Norwegian hospital in 2018-2019. Interview transcripts were subjected to thematic content analysis. In this paper, we focus on the subject of nonuse of PAEHRs.

**Results:**

We identified 8 different rationales that study participants had for not embracing PAEHRs. When patients reflected on why they might not use PAEHRs, they variously explained that they found PAEHRs unnecessary (they did not feel they were useful), impersonal (they preferred oral dialogue with their doctor or nurse over written information), incomprehensible (the records contained medical terminology and explanations that were hard to understand), misery oriented (the records solely focused on disease), fear provoking (reading the records could cause unwanted emotional reactions), energy demanding (making sense of the records added to the work of being a patient), cumbersome (especially among patients who felt they did not have the necessary digital competence), and impoverishing (they were skeptical about the digital transformation of individual and social life).

**Conclusions:**

It is often assumed that the barriers to PAEHR use are mostly practical (such as lack of hardware and access to the internet). In this study, we showed that patients may have many other valid reasons for not wanting to adopt this kind of service. The results can help guide how PAEHRs and other digital health services are promoted and presented to patients, and they may suggest that the goal of a given new digital health service should not necessarily be full uptake by all patients. Rather, one should recognize that different patients might prefer and benefit from different kinds of “analog” and digital health services.

## Introduction

### Background

Over the past decade, patient-accessible electronic health records (PAEHRs) have been requested, developed, introduced, and advocated for in health care systems in several nations [1,2]. The existing literature conveys the impression that a majority of patients are satisfied with PAEHRs and argue that they can increase patient involvement, improve patient-provider communication, support self-management, and promote patient empowerment [3-6]. In a survey of patient experiences in Norway, the majority of users were satisfied overall with PAEHRs [7] and perceived online access to their medical records as useful for tasks such as looking up health information, keeping track of ongoing treatments, preparing for medical appointments, and sharing medical documentation with others. Similar findings have been reported from other countries [8-10].

Notwithstanding this, there are always significant minorities of patients in PAEHR surveys who are less enthusiastic about PAEHRs. For example, 6% of respondents in a Swedish survey indicated that they did not think PAEHRs led to better understanding [8], 12% of respondents in a US survey were concerned about their privacy in connection with PAEHR use [9], and 19% of respondents in the Norwegian study mentioned above had become worried because of information they found in their health records [7].

Little is known about the perspectives of patients who do not access their records. A qualitative study from Sweden included 15 patients with cancer without experience using PAEHRs [11]. The main argument of these patients for not accessing records was reported to be “that they have a good relationship with their physician and that they receive the information they need,” but their rationales for not using the service were not explored further. A better understanding of why patients may resist using new digital health services like PAEHRs is important for further successful development and implementation of digital services.

### Digitalization in Norway

Compared with many other countries, Norway has high coverage of internet access and use, with 98% of the population aged between 16 and 79 years having used the internet for different purposes during the last 3 months [12]. A range of different public and commercial services have been extensively digitalized (eg, banking—to the degree that it is now highly inconvenient to handle personal banking outside the web), and most citizens employ digital tools in their everyday lives [13]. Therefore, we saw Norway as a good setting for studying patients’ views on electronic health services that go beyond issues concerning the digital divide.

Providing all patients with electronic access to their health records has been a goal for the Norwegian government since 2012 [14]. A white paper entitled “En innbygger, en journal” (“One citizen, one health record”) argued that such access “gives citizens overview of their own information and knowledge about their health and illness. This provides a basis for co-decision making and active cooperation between health care professionals and patients and clients. Patients and clients will, through access and participation, be able to point out errors in the health records and improve the quality of the documentation” [14].

### “Pasientjournal”: The PAEHR Used in Norwegian Specialized Health Care

In December 2015, PAEHRs were introduced under the name Pasientjournal by the publicly owned Northern Norway Regional Health Authority, the owner of the hospital in which fieldwork for this study was carried out. The stated goal was to “increase patient empowerment and patients’ involvement in their own health, and improve the quality of services” [15]. In a video promoting the service, the Pasientjournal is described as a tool for becoming an “enlightened patient” [16].

The Pasientjournal service is now available to anyone seeking public specialist health care in 3 of Norway’s 4 health regions, which cover roughly 80% of the Norwegian population. It gives patients access to almost all documents in their medical records. They can retrieve and read doctors’ notes, discharge notes, outpatient clinic notes, nurses’ documentation, physiotherapy notes, referral notes, radiology reports, and some pathology test results. Laboratory results are not yet available but will be in the near future. All documents are accessible by the patient as soon as they have been digitally signed by the responsible health care professional. The list of documents is ordered chronologically and can comprise a few entries to hundreds of entries depending on how much contact the patient has had with health care services. Patients can click on any given document to read its full contents. Apart from reading, the patient cannot take any other action online. If one has a question or discovers something incorrect, one is encouraged to contact the hospital for further guidance. Patients log onto the service using the same identification procedure as for several other public digital services.

Based on qualitative research in a Norwegian hospital, the aim of this paper was to explore what patients conceive of as the main weaknesses and disadvantages of PAEHRs as well as their rationales for not actively embracing online access to their health records. We based our analysis on interviews with PAEHR users (both regular and occasional) as well as with patients who had never accessed their records online.

## Methods

The fieldwork for this study was carried out in 2018-2019 in a hospital owned by the Northern Norway Regional Health Authority. We have decided to conceal the name of the hospital as part of our efforts to protect study participants’ personal information.

### Study Design

This paper draws on a study that was inspired by the ethnographic research tradition. The first author (HSV) did fieldwork for a period of 12 weeks in the hospital’s department of internal medicine, which consists of several wards and outpatient clinics and covers a range of medical specialties (nephrology, endocrinology, cardiology, pulmonology, gastroenterology, hematology, and oncology). Fieldwork entailed participant observation as well as semistructured interviewing of patients and health care professionals, with the overarching goal of exploring how PAEHRs are incorporated into everyday practices in a hospital. This paper is based on an analysis of the patient interviews conducted in the study.

### Qualitative Interviews and Recruitment of Study Participants

A total of 40 patients were interviewed. They were recruited in the course of participant observation, during which the first author (HSV) accompanied doctors and nurses while they were doing their daily work in the department. Patients who gave their consent were invited to take part in an interview. The researcher had no knowledge of the invited patients’ experience with PAEHRs prior to inviting them to participate in the study, unless this had become evident in the observed interaction with their doctor or nurse. To help ensure that patients were competent to give consent and in a condition that allowed them to sit through an interview, potential study participants were discussed with their care providers before they were invited to participate in the study. All but one of the patients who were asked to participate accepted the invitation. They received information about the study both orally and in writing before they were asked to provide written consent to participate.

Patients were recruited as the first author moved around the clinic and aimed to include patients with a variety of ages, genders, and diagnoses. The variety was obtained by recruiting patients from both the inpatient and the outpatient clinic and by following health care professionals from different medical specialties. Of the 40 interviewees, 22 were women and 18 were men. Their ages varied between 21 and 84 years. The study participants had a range of different medical conditions in the domains of nephrology, cardiology, cancer, hematology, lung diseases, and infections. Among the 40 interviewees, 32 knew about Pasientjournal prior to the interview and 8 did not. Among the latter, 6 indicated that they felt positive about the idea of logging into and reading their medical records while 2 did not. A little less than half (ie, 16 individuals) had used PAEHRs either occasionally or regularly prior to the interview.

The interviews, which lasted between 15 and 60 minutes, took place by the hospital bed in the case of admitted patients and in offices and examination rooms in the case of outpatients. An interview guide listing topics to be covered (eg, the patient’s history of illness, prior knowledge and use of the PAEHR, health information–seeking habits, and other digital habits) was used as a memory aid during the conversations, but participants were encouraged to speak freely and the interviewer sought to let the participants take the lead in the dialogue. The interviewer’s aim was to be a conversation partner who traveled along with the patient into their stories rather than someone trying to “dig out” information from them (this style of interviewing was inspired by Brinkmann and Kvale’s metaphor of the interviewer as a “traveler” [17]).

In this paper, we draw on the data generated through qualitative interviewing, focusing specifically on patients’ rationales for not using PAEHRs. We plan to return to other study findings in future works.

### Data Analysis

All interviews were transcribed verbatim, and transcripts were read carefully several times. Following a stepwise deductive-inductive approach [18], we then coded the interviews empirically close with in vivo coding, meaning that many of the codes contained words and expressions used by the study participants themselves. The initial coding was done by the first author and then discussed thoroughly with the coauthors. In a subsequent step, codes were grouped into themes. Identified themes were discussed and reordered during several analysis meetings involving all authors until a common understanding had been reached. For the purpose of this paper, themes relating to why patients do or do not use PAEHRs were chosen and further analyzed. The themes identified in this article were generated on the basis of interviews with patients who did not want to use the PAEHR (in spite of knowledge of the service) as well as with patients who had personal experience with Pasientjournal.

### Researcher Positions

Fieldwork was conducted by the first author (HSV). She is a woman in her 30s who is a trained physician and has clinical experience from work in hospitals (but she had not worked in a clinical setting for several years when fieldwork started and had not worked in a clinical setting after PAEHRs were introduced). She also has personal experience as a close relative of severely ill patients. Her medical background made it easy to move around in the clinic, as many hospital “codes” and routines were well known to her. She made efforts to explain to study participants that she was there as a researcher and not as a representative of the hospital management. At the hospital’s request, she dressed in white scrubs during fieldwork. She thereby blended in with the staff and at times had to underline for the study participants that she was not present by virtue of her medical skills.

The second and third authors (AKL and KM, respectively) are also trained physicians, but both currently work full time as associate professors (in the fields of medical anthropology and medical history, respectively).

All authors engage with many digital tools in their personal and work lives and view themselves as highly computer literate and often appreciate digital solutions. None of the authors had clear opinions on PAEHRs prior to the study. They entered the study with curiosity and a wish to better understand patient experiences with PAEHR.

### Ethical Considerations

The study was approved by the Norwegian Data Protection Authority (case no. 56987). Exemption from health care workers’ duty to maintain confidentiality was obtained from the Regional Committee for Medical and Health Research. In the hospital, fieldwork was approved by the head of the clinic, the chief of research, and the data protection officer. All study participants gave informed written consent to participate and were told that they could withdraw from the study at any time without giving a reason. Directly person-identifying information were omitted in transcripts, and audio recordings were deleted after transcription. A key that linked the data to participants’ names and contact information was kept in a separate and securely locked location. All data were stored and processed in “Service for Sensitive Data” (Tjeneste for Sensitive Data), University of Oslo’s platform for collecting, storing, analyzing, and sharing sensitive data in compliance with the Norwegian privacy regulations.

## Results

The study participants had a range of different rationales both for and against the use of Pasientjournal. In this paper, our focus was on the latter, and in this section, we present the 8 different rationales against the use of Pasientjournal that we identified in the course of the study.

### Unnecessary: No Need to Access the Health Records

One rationale against the use of PAEHRs was that the digital health records seemed unnecessary. For some of the study participants who had never accessed their PAEHR, the question was clearly not why they did not use the service but rather why they would ever do such a thing. They struggled to see how they could benefit from accessing Pasientjournal. Many felt they got the information they needed from their doctors and trusted them to provide necessary guidance and information. Some questioned how reading the records would make a difference for their health. For example, a woman in her 70s stated the following when the first author tried to dig into the reason behind her lack of interest in the PAEHR: “No, I do not believe that it would make me healthier” [Patient 12, a woman in her 70s; no PAEHR experience].

On the ward, paper copies of discharge notes were routinely given to all patients at departure. Many study participants felt that this was all the written information they needed:

"I don’t know, really. The times I’ve been admitted, I’ve usually gotten the discharge note handed out, so I have not, like, had any reason to ... or felt a need for it. I have been given good information from the doctors I have been seeing as well"

Patient 22, a woman in her 50s; no PAEHR experience

In summary, some study participants had not felt motivated to spend time accessing the PAEHR, and they often argued that they had felt satisfied with the information they had been provided orally and in written discharge notes.

### Impersonal: Prefer Dialogue to Information

Some study participants not only thought that information received in dialogue with health care workers was “good enough” but reasoned that it was superior to the information accessible through PAEHRs. Patient 14, for example, pointed out that medical records do not allow you to ask anyone the questions that pop up while you are reading and for that reason saw PAEHRs as inferior to face-to-face communication:

"Though I probably would get the same information, but you do not get to ask the questions you might have there and then, or get it explained if there is something you find hard to understand. I do think it is better to speak to someone about it than just reading it!"

Patient 14, a woman in her 40s; PAEHR user

A bit worried, she went on to ask if there was an intention to replace physical meetings with this form of written information, and added, “If so, I would see that as a loss.”

For some, the thought of logging onto a computer to access information about one’s own health seemed rather soulless. For Patient 11, for example, the human contact in face-to-face communication had considerable value:

"I’m a very verbal kind of person. If there is something that really annoys me, at work for example; the times where you are expected to find all the information for yourself, instead of just meeting up in the hallway and I can say: Let’s meet up after lunch and discuss this. So I really like that human contact, and sometimes it’s kind of a principle for me to pursue that line."

Patient 11, a man in his 40s; PAEHR user

A common finding throughout the fieldwork was that patients’ use of superlatives in conversations about health care was typically related to individuals who had made a positive impression. Many praised individual physicians and nurses who had made a difference for them (although some, of course, also told of negative experiences). The same type of superlatives was hardly ever used about PAEHR.

### Incomprehensible: Patients Are Not the Intended Reader of the Medical Records

Another reason why PAEHRs were not always perceived as attractive was that the language used in medical records could be hard to understand. Patient 7, for example, a retired cleaner, had never logged into Pasientjournal for the very reason that she did not think she would understand much of the contents:

"It has to be understandable, so they must not use a lot of foreign words that I don’t know or Latin words for different things, this and that and that"

Patient 7, a woman in her 60s; no PAEHR experience

Patient 32 expressed a similar sentiment, even though she worked as an auxiliary nurse. When asked if she knew she could access her records, she answered:

"Yes, I do know that, but I am thinking: What’s the point? The doctors’ language ... I certainly do not understand much of that."

Patient 32, a woman in her 50s; no PAEHR experience

Many of the participants who had accessed their records also described how the medical records could be difficult to comprehend. Patient 10, for example, had accessed Pasientjournal to see what her doctor had written about the results of a sleep monitoring procedure she had undergone. She found the note, but she felt she could not really make much sense of it:

"You know, many of the documents in there, they are more like letters to doctors, or therapists, so there is a lot of inexplicable words, and I do not understand everything, I really don’t."

Patient 10, a woman in her 20s; PAEHR user

### Misery Oriented: Too Much Focus on Disease

Several study participants had reservations about PAEHRs because they wanted to avoid unnecessary focus on ill health. Patient 3, for example, a man in his 60s who had a chronic condition that demanded regular blood transfusions, explained it this way:

"I wouldn’t say that I am totally uninterested in my disease; of course I’m not. But I’m not all that interested in it either, as long as everything is working as well as it does. So, I have chosen not to think about this disease too much. ... I think it is important for the total well-being, or the feeling of not digging oneself down in the disease and give it your main focus. I focus on all the things I can do, and that’s a whole lot of things. ... I believe that it is important to be able to live with this kind of serious, chronic disease; that you do not go around thinking about it all the time. I believe that will make you feel like you are locked in inside yourself."

Patient 3, a man in his 60s; no PAEHR experience

Similarly, Patient 32, a woman in her 50s, also questioned what good might come from worrying about things she could not do anything about:

"You see, what good will it do to anyone around me if I were to dig down in my own misery? If this is something I will die from, then I want to live while I’m alive"

Patient 32, a woman in her 50s; no PAEHR experience

What these participants seemed to argue was that immersing oneself in disease and medical knowledge may not be a favorable way of dealing with illness and might even stand in the way of “good health.”

### Fear Provoking: Promoting Unnecessary Worry

Some study participants were concerned that reading the medical records would make them upset or worried. In their assessment, accessing their records had the potential to harm.

"No, if one starts to follow up on that stuff, one will start to speculate about all sorts of things, and then it certainly becomes worse. ... that is also why I never Google survival outcomes on stenting of heart vessels, that’s for sure!"

Patient 13, a man in his 50s; no PAEHR experience

As Patient 15 pointed out,

"In some ways it feels good to be kind of ignorant, too. I do not want to read any reports before I know that this [the disease] has turned out good, in a way."

Patient 15, a man in his 40s; no PAEHR experience

Some participants who had used Pasientjournal explained how reading the health records had led to negative feelings. For example, a man suffering from incurable cancer explained how reading about his prognosis felt harder and more brutal than talking to his doctor about it:

"Even if it is information that I already know, it’s sort of hard at times to sit down and read, when it’s there, black on white, that they [the doctors] are not that optimistic anymore, you know. ... so it’s kind of brutal."

Patient 4, a man in his 50s; PAEHR user

Avoiding the worries and negative feelings that they believed could result from being exposed to the illness focus in the health records constituted an important rationale for some of the participants for not engaging with the PAEHR.

### Energy Demanding: Unsuitable When Ill Health Drains You of Your Vigor

When experiencing serious disease, patients may be indisposed of their normal capacities. Under such circumstances, some of the study participants had simply not had the energy to log onto Pasientjournal. In the case of Patient 5, this phase had lasted for several months after she was diagnosed with and went through surgery for breast cancer. Although she knew about the PAEHR and thought of it as an interesting service, she had not yet had the energy to use it 3 months after her surgery:

"I have read about that thing, that it is a possibility, but it has been so much information to digest and absorb that I did not reckon I had the time to log on there. ... have just been so overwhelmed with thoughts, with this disease, so I have just not had the energy"

Patient 5, a woman in her 40s; no PAEHR experience

Participants who had experienced serious disease talked about the overwhelming situation they suddenly had found themselves in when going from being healthy and resourceful to becoming a patient. Some also described how being a patient involved a lot of work. Patient 1, for instance, had just retired when she was diagnosed with cancer. She described how dealing with her new situation felt like a job with lots of tasks to take on:

"Everything was just so unfamiliar, and I did not know how to handle things when I became ill, and all the stuff that came along with the disease. ...This [being sick], it is actually a full-time job. With added extra hours!"

Patient 1, a woman in her 60s; no PAEHR experience

The “energy demanding” rationale against PAEHR use draws from the experience of illness as something that demands effort from the patient in many different ways. In such situations, the task of reading health records may be too much to deal with on top of everything else.

### Cumbersome: Screen May Not Be Better Than Paper

PAEHR solutions make health records available to patients through the internet. While this is often touted as making information “easily available,” some study participants felt that electronic access was a cumbersome thing. Some, especially in the older age groups, explained how navigating and engaging with new digital services could be an onerous task. Although many used personal computers and smartphones for other undertakings, they could find themselves struggling when they had to handle a new service on their own. Most patients had relatives who they turned to with “data troubles,” but some felt uneasy bothering them too much. Patient 37, who regularly turned to her son for help with “technical stuff,” explained:

"You know, my son, he is an expert on data, so he tries to explain it to me: ‘Mother, you have to click here, and then this and that,’ but then he has to run to catch the bus, and I’m like: ‘What did he say???’ Even before he’s out the door, it’s all gone for me."

Patient 37, a woman in her 70s; no PAEHR experience

Among the participants who questioned the “easiness” of digital solutions was Patient 12, a woman in her 70s. She had become upset when her pharmacy stopped giving out lists of prescriptions on paper and instead encouraged its customers to go online. She had logged on several times to check her prescriptions, but when the pharmacy did away with the new policy, she was relieved. Although she mastered the digital tool and could access her prescriptions, she found it much less complicated to be given a paper copy when she picked up her medicines. Patient 37 and Patient 12 are examples of patients who do not find that digital access represents progress and is “easier” than analog solutions.

A consequence of needing assistance to use a patient portal is that one must be willing to let the helper see the contents of one’s medical records. Some study participants were not comfortable with this, and one explained that this was the main reason why she had never used the Pasientjournal.

### Impoverishing: Resisting the Digital Transformation of Individual and Social Life

A final rationale against the use of PAEHRs was a general skepticism toward the ongoing digitalization of life worlds. Patient 7, for example—a woman in her 70s who was admitted to the hospital with newly discovered cancer—explained that she felt frustrated by how everyone was hooked on their screens and how this affected the social life in her family.

"I don’t want to get into that stuff. I see everyone sitting there with their tablets all the time; they are completely absorbed. You lose the social part, I’ve said that from the beginning. And it’s all...and it includes the kids as well, and the grandkids. They are all staring into a tablet, they all have a phone they are constantly playing on, or something. And I really resent much of that! Because you don’t have the social engagement anymore."

Patient 7, a woman in her 70s; no PAEHR experience

Patient 7 had chosen not to engage in any digital affairs. She left that to her husband. They had a computer at home, but she never used it. “I’m not at all interested in that!” she stated, and she did not see the PAEHR as a service worth embracing. Patient 7 was the most prominent representative of an antidigital attitude in our study.

Other study participants were less vigorous in their rejection of digitalization but still expressed that they wanted to reduce the time spent in front of screens and computers, limiting such activities to strictly necessary chores. Patient 38, a man in his 70s, had heard of PAEHRs but had not yet used Pasientjournal. When asked if he would consider accessing his hospital health records, he was a bit hesitant, and said, “I guess I could do that.” When pushed a bit further, he explained how he had experienced that computers interfered with his former work life as a shop owner:

"It took me away from the clients. Instead of being out in the shop and talking to customers, I was stuck at the back dealing with computer issues and troubles."

Patient 38, a man in his 70s; no PAEHR experience

Based on his experiences, Patient 38 felt a reluctance to engage too much with computers.

## Discussion

### Principal Findings

In this study into patients’ rationales for not embracing online access to health records, we identified 8 main motivations for nonuse. These results expand the current knowledge of patients’ perspectives on PAEHRs.

A fundamental reason why some study participants did not embrace PAEHRs in our study was that they did not think that reading their own medical record was particularly useful. It was unclear to them exactly what, if anything, reading medical notes would add to their understanding of their ailment, medical care, and/or condition. These views are in contrast to the more positive perspectives on the utility of PAEHRs reported in several previous studies [7,11] as well as by many of the participants with user experience in this study (to be published in another article). Participants who fronted the “unnecessary” rationale against PAEHRs felt that they got the necessary information from their doctors and did not seem to desire more medical information.

Several patients also preferred dialogic information given face to face rather than written information, which brings us to the second rationale identified: medical records are impersonal. Reading PAEHRs is an activity that does not typically involve human contact or interaction, and PAEHRs provide no 2-way interaction. As you read, there is no one to ask, no one to laugh or cry with, and no one to seek comfort and reassurance from. If considerations related to efficiency and costs should lead to human interaction being substituted by digital access to records that are not written primarily with the patient in mind, health care would surely risk becoming impoverished.

Many of our study participants (albeit not all) felt that their health care services were well-functioning and that their need for information and explanation was met in direct communication with their health care providers. One could hypothesize that PAEHRs might be perceived of as relatively more useful in a setting where patients were not as content with the services and the information they received. There may be some evidence to support this hypothesis. Surveys of PAEHR users in the United States have found that patients from underserved groups (less educated, non-White, older, and Hispanic patients, and individuals for whom English is a second language) are the ones most likely to report “major benefits” from reading medical notes [9].

An issue raised by study participants who had never used PAEHRs, as well as those who had used it either frequently or infrequently, was that laypeople are not the intended audience for the documents accessible through PAEHRs. A survey of Norwegian PAEHR users previously found that understanding the content of the medical records is a challenge for many: 36% indicated that it was hard to understand what the documents were about, and 59% reported that they had difficulties understanding the medical terminology [7]. A Danish study that examined the language in 10 medical records with the aim of identifying “potential lay-friendliness, patient-centeredness, and patient empowerment” found that the records were written in highly specialized expert language dominated by expert terminology, expert syntax, expert presuppositions, and difficult abbreviations [19]. The authors concluded that “the majority of Danes will not fully understand their own e-records and will have a high potential for misunderstandings” [19]. In sum, few medical record entries are written with patients as the intended reader. Notes are typically prepared with other functions in mind and tend to be worded with the understanding that the medical file is a communication platform for professionals engaged in the care of the patient. The logic behind assessments, choices, and treatments is seldom explained explicitly; the text rests on the expectation that the reader is a health care professional with certain crucial preunderstandings. In short, the health record is not a resource dedicated to providing enlightenment, sympathy, or education to patients. Of course, this is not to say that the information in the records are never useful for patients, and many patients see PAEHRs as a good place to find information. Other studies have found that some patients use the PAEHR to remember what they talked about with their doctor or to prepare for an upcoming doctor’s appointment [20]. However, as described in this study, the unfamiliar and professional atmosphere in the health record served as a barrier to PAEHR use for some patients, and they preferred using other sources for information about their health and illness. In the future, this barrier may be lowered by tools providing easy translation and explanations of medical terminology used in the PAEHR [21]. It must also be pointed out that Pasientjournal is currently only available within specialized health care services, and therefore the contents in the health records are often of a specialized and rather complex nature. One could hypothesize that the content of the records kept in general practice might be of a more accessible and understandable nature, and that the “incomprehensible” rationale might be less prominent in general practice settings than in hospitals.

Medical records may shift one’s focus from healthy and wholesome parts of life to sickness and worries about ill health. Avoiding unnecessary focus on sickness and pathology seemed to be an important strategy for some of our study participants. Similar findings were described in a qualitative study of self-management practices among patients with chronic conditions. Van de Bovenkamp and Dwarswaard [22] explained how “self-management is very much shaped by patients’ ideas of the good life.” A study by Henwood et al [23] of health information–seeking practices among women in midlife similarly identified resistance against the idea that patients should seek out medical information by themselves rather than trusting their doctors to equip them with it. The quotation used by the authors in the article’s title—”ignorance is bliss sometimes”—resonates with some of the patients’ views in this study: more knowledge is not necessarily for the better. Moreover, some of our study participants clearly also actively resisted the biomedical version of their conditions as presented in the medical records [24]. To them, illness was more than the biomedically relevant information written down in the PAEHR. Illness included feelings, everyday challenges, and encouragements—in short, components of a life that the biomedically dominated health records were lacking.

In addition, some participants stayed away from PAEHRs because they felt that medical records might have the capacity to induce fear and anxiety, and some described situations in which reading the medical records had led to unwanted emotional reactions. This is reflected in a Norwegian survey of PAEHR users as well: 20% of respondents indicated that they had become worried by reading health records [7]. Clearly, how much and what kind of information one seeks at what time will depend on individual factors and previous experience and may also change over time. Some PAEHR nonusers preferred to stay somewhat “ignorant” and rely on the doctor to curate the necessary information for them.

The “new patient role” has been critiqued for not recognizing that patients oftentimes are unable and weakened—temporarily or permanently—when they are sick, and that this affects their ability to act as they otherwise would and make “rational” choices [25,26]. Reading and making sense of the health records is a complicated task (at times even for health professionals). It requires concentration over time and cognitive capacity to absorb information. Lack of concentration can be a temporary or enduring result of a patient’s condition or a side effect of medication—all described by participants in this study. For some of the interviewees, this was exactly why they had not accessed their health records. Moreover, as also described by participants in this study, becoming a patient may resemble taking on a new full-time job. As Mol argued in *The Logic of Care* [26], the notion of the neoliberal “patient citizen” (or “active patient” in the PAEHR context) presupposes a nonsuffering person who can make well-argued individual choices, whereas in reality the patient is often weakened by disease and emotional distress that delimits his or her ordinary capacities. The experiences of our participants related to Mol’s argument that good care implies engagement in ongoing attempts to adapt knowledge and technologies in a collaboration involving patients and their health care providers as well as their diseased bodies and complex lives.

Certainly, technological obstacles also played a role in our study. To make use of PAEHRs, some of our participants felt they would have to ask for help from relatives or friends, thereby revealing the dependency situation digital health records may impose on patients. Wyatt et al [27] have described how using the internet for health information seeking may require access to competent helpers that can guide less-experienced users in finding and making sense of the information. This was also a concern for some of the participants in this study, and perhaps not surprisingly, especially among some of those who were older. Several participants used the internet for other purposes but were not confident they could handle a new service or task without assistance. Many were hesitant to ask for help from relatives or friends who were more digitally competent (“warm experts,” to use Bakardjieva’s term [28]). Depending on the nature of a person’s current and former health-related issues, the record can contain information that patients feel is sensitive and too private to share with others, even (and sometimes especially) with close relatives. The threshold to ask for help from “warm experts” might therefore be higher in the case of PAEHRs than for many other digital tasks.

Finally, some of the study participants were generally critical toward the digitalization of individual and social life. In a world characterized by optimism on behalf of digital innovations, this kind of general resistance is at times dismissed as reactionary, and its representatives may at times seem to be rendered invisible in public discourse. However, our study revealed sound and rational arguments for staying outside of digital life. In health care services, this group must also be taken into account to ensure that all patients are equally taken care of within the health care system.

### Limitations and Further Research

This article builds on qualitative research and we are therefore in no position to know how common the rationales against PAEHR use identified by our study participants may be. Moreover, our study was limited in time, geography, and size, and further work in the same location or elsewhere could bring out additional rationales of relevance and importance. Furthermore, there is a potential for researcher bias here as we know that many health personnel are skeptical toward PEAHRs and the 3 authors are all physicians. However, all 3 of us have chosen other careers than the clinical as our main jobs and do not primarily identify as clinicians. None of us had preconceived ideas of a potential negative impact before embarking on this study.

There is a need for more research on rationales for not using PAEHRs. Surveys quantifying different rationales for and against PAEHR use, as well as studies of the demography of users and nonusers, are important. There is also a need for more qualitative studies exploring potential differences between specialist health care settings and primary health care settings, as well as possible factors contributing to positive outcomes from and experiences with PAEHRs.

### Practical Implications

This study demonstrates that patients may have good reasons for not using PAEHRs and could help guide how PAEHRs are promoted among patients. While PAEHRs are a beneficial service for many patients, it may not be the case for all patients. Health care providers should take this into consideration and should avoid assuming that information is received or understood because it is available via PAEHRs. Moreover, when PAEHRs are promoted, one should avoid making the assumption that online access to health records will be useful for all or should be used by all.

### Conclusions

It is often assumed that barriers against PAEHR use are mostly practical (such as lack of access to hardware, slow internet connections, etc) and that once such hurdles are removed, PAEHRs will be widely embraced. In this paper, however, we have demonstrated that patients may have many good reasons not to celebrate or adopt this kind of service. Assessed as a potential source of insight and understanding, some find that PAEHRs are less than perfect because they are devoid of human interaction and contact, use language that they do not understand, promote a 1-sided focus on misery, may lead to unnecessary fear, demand more energy than one may have when sick, threaten to expose one’s secrets, and can be cumbersome to make work. Perhaps more than anything else, what the study participants highlighted was that the information in health records is not in a format that is genuinely written for patients. To the contrary, health records are primarily a peephole into biomedical perspectives on the patient’s misfortune and ill health. What one gets access to through PAEHRs is often biomedical explanatory models written in biomedical language for biomedically trained professionals. This study demonstrates that not all patients view this as either helpful or favorable, and promoting the view that PAEHRs are something all patients ought to engage with might lead some patients to feel pushed into a domain they are not comfortable in.

## Abbreviations

PAEHR: patient-accessible electronic health record
